# Supplementary material for: A Likelihood Approach for Real-Time Calibration of Stochastic Compartmental Epidemic Models
Source: PLoS Comput Biol. 2017 Jan 17;13(1):e1005257. doi: 10.1371/journal.pcbi.1005257 (PMC5240920; doi:10.1371/journal.pcbi.1005257)
Supplement: S1 File — (TAR.GZ) [file pcbi.1005257.s014.tar.gz › HSPH_Online-SI-Revision/EnKF07/n100-mild/n100-mild_EnKF2_table8.pdf]

1	newly 3 weeks spec	114.	1289.15	{17., 3105.}	1079.5	0.684305	newly 3 weeks cumul	602.	13 456.5	{3348., 44 860.}	2138.54	1.12416	newly 5 weeks spec	30.	510.75	{0., 256.}	1721.57	1.54965	newly 5 weeks cumul	708.	14 743.9	{3984., 48 688.}	1985.82	1.0861	newly 44 weeks cumul	814.	15 671.2	{4384., 51 357.}	1828.58	1.04392
2	newly 3 weeks spec	204.	798.63	{0., 969.}	380.946	1.31836	newly 3 weeks cumul	827.	6432.11	{2990., 6459.}	680.874	0.738895	newly 5 weeks spec	99.	318.7	{0., 588.}	357.717	1.71997	newly 5 weeks cumul	1036.	7241.13	{3797., 7005.}	602.067	0.679702	newly 43 weeks cumul	1187.	8043.48	{3797., 7445.}	580.398	0.656369
3	newly 3 weeks spec	108.	2501.71	{257., 12 667.}	2216.4	0.775208	newly 3 weeks cumul	712.	8822.9	{1749., 44 505.}	1139.17	0.655865	newly 5 weeks spec	44.	1534.31	{62., 6544.}	3389.2	0.78982	newly 5 weeks cumul	820.	12 345.6	{2225., 65 367.}	1405.56	0.690452	newly 46 weeks cumul	845.	17 078.3	{2420., 81 993.}	1921.1	0.762836
4	newly 3 weeks spec	19.	900.91	{10., 996.}	4657.74	1.18552	newly 3 weeks cumul	149.	3974.09	{103., 4856.}	2581.7	1.21525	newly 5 weeks spec	0.1	565.95	{0., 507.}	565 876.	2.69237	newly 5 weeks cumul	155.	5247.58	{139., 6586.}	3296.67	1.26066	newly 50 weeks cumul	155.	8142.18	{190., 9690.}	5161.54	1.32974
5	newly 3 weeks spec	147.	1077.86	{251., 784.}	633.265	0.540039	newly 3 weeks cumul	785.	3777.47	{1576., 2804.}	381.206	0.469203	newly 5 weeks spec	78.	806.17	{61., 871.}	944.756	0.566077	newly 5 weeks cumul	977.	5523.59	{2033., 4825.}	465.362	0.495507	newly 46 weeks cumul	1144.	10 105.5	{2101., 13 755.}	783.344	0.576258
6	newly 3 weeks spec	116.	2444.44	{55., 7577.}	2027.67	1.07955	newly 3 weeks cumul	649.	16 487.9	{1322., 51 296.}	2442.5	1.11536	newly 5 weeks spec	30.	596.03	{3., 830.}	1918.57	1.20973	newly 5 weeks cumul	747.	18 225.7	{1346., 54 239.}	2342.01	1.10144	newly 44 weeks cumul	826.	19 014.8	{1346., 54 438.}	2203.94	1.07465
7	newly 3 weeks spec	14.	1211.86	{95., 4296.}	8556.14	1.48659	newly 3 weeks cumul	126.	4183.26	{289., 13 506.}	3220.05	1.11179	newly 5 weeks spec	0.1	864.54	{78., 4191.}	864 440.	3.43012	newly 5 weeks cumul	128.	6081.9	{445., 20 830.}	4651.48	1.25426	newly 49 weeks cumul	128.	11 189.1	{1250., 43 806.}	8641.47	1.48194
8	newly 3 weeks spec	39.	1786.11	{203., 4319.}	4479.77	1.30139	newly 3 weeks cumul	302.	11 524.1	{3419., 29 515.}	3715.94	1.36764	newly 5 weeks spec	15.	611.2	{12., 1465.}	3988.8	1.07696	newly 5 weeks cumul	333.	13 162.1	{3743., 33 107.}	3852.59	1.36971	newly 43 weeks cumul	361.	14 159.7	{3846., 33 369.}	3822.37	1.35711
9	newly 3 weeks spec	85.	3915.94	{208., 9666.}	4507.84	1.22866	newly 3 weeks cumul	398.	13 151.7	{1457., 25 460.}	3205.57	1.17334	newly 5 weeks spec	41.	2921.26	{110., 11 257.}	7028.24	1.28578	newly 5 weeks cumul	509.	19 460.4	{2203., 47 386.}	3724.25	1.20951	newly 48 weeks cumul	521.	30 742.3	{3305., 98 102.}	5801.35	1.3489
10	newly 3 weeks spec	178.	579.08	{35., 1065.}	280.618	0.516727	newly 3 weeks cumul	763.	13 233.	{3674., 6780.}	1635.02	0.852308	newly 5 weeks spec	52.	49.67	{0., 100.}	102.596	1.02703	newly 5 weeks cumul	913.	13 437.7	{3682., 7463.}	1373.1	0.789331	newly 42 weeks cumul	1004.	13 471.2	{3684., 7834.}	1243.46	0.752757
11	newly 3 weeks spec	68.	1601.82	{391., 1123.}	2255.62	1.06567	newly 3 weeks cumul	409.	6585.79	{2647., 4885.}	1510.22	0.978195	newly 5 weeks spec	9.	982.2	{83., 1256.}	10 813.3	1.5642	newly 5 weeks cumul	430.	8795.37	{3247., 6187.}	1945.43	1.06317	newly 46 weeks cumul	447.	14 535.4	{3503., 20 863.}	3151.77	1.17597
12	newly 3 weeks spec	387.	749.59	{0., 1435.}	205.269	1.90798	newly 3 weeks cumul	1436.	10 612.4	{3431., 15 071.}	639.022	0.638451	newly 5 weeks spec	224.	141.83	{0., 381.}	105.754	2.0499	newly 5 weeks cumul	1967.	11 075.6	{3444., 15 075.}	463.068	0.520422	newly 42 weeks cumul	2777.	11 222.	{3444., 15 075.}	304.104	0.377405
13	newly 3 weeks spec	44.	3245.11	{303., 19 292.}	7275.25	1.43274	newly 3 weeks cumul	425.	14 345.7	{4169., 55 441.}	3275.65	1.27765	newly 5 weeks spec	11.	764.71	{44., 1839.}	6852.45	1.37986	newly 5 weeks cumul	440.	16 725.1	{4892., 82 044.}	3701.16	1.31492	newly 47 weeks cumul	442.	17 445.4	{5227., 91 174.}	3846.92	1.33317
14	newly 3 weeks spec	73.	1037.47	{10., 4246.}	1379.38	1.07787	newly 3 weeks cumul	262.	5927.57	{435., 19 835.}	2172.44	1.03347	newly 5 weeks spec	18.	418.14	{0., 821.}	2299.22	1.47367	newly 5 weeks cumul	346.	6992.65	{436., 23 102.}	1931.44	0.971193	newly 49 weeks cumul	354.	7841.3	{436., 24 076.}	2125.53	0.98921
15	newly 3 weeks spec	78.	2304.39	{298., 1341.}	2855.53	0.965634	newly 3 weeks cumul	442.	8651.	{1032., 6465.}	1859.23	0.928953	newly 5 weeks spec	22.	1469.45	{42., 981.}	6580.05	1.09433	newly 5 weeks cumul	504.	11 963.4	{1548., 7703.}	2275.37	0.974273	newly 46 weeks cumul	595.	16 355.4	{3078., 13 067.}	2650.36	1.01362
16	newly 3 weeks spec	250.	245.58	{0., 603.}	127.864	2.22682	newly 3 weeks cumul	1232.	5456.06	{3660., 5444.}	343.078	0.584603	newly 5 weeks spec	117.	42.62	{0., 136.}	83.8803	2.06877	newly 5 weeks cumul	1525.	5600.79	{3820., 5568.}	267.855	0.505251	newly 42 weeks cumul	1724.	5634.9	{3826., 5580.}	227.693	0.456597
17	newly 3 weeks spec	47.	2374.23	{0., 9041.}	4999.34	1.55497	newly 3 weeks cumul	382.	15 182.2	{651., 51 959.}	3874.95	1.11965	newly 5 weeks spec	36.	1926.73	{0., 10 742.}	5302.03	1.54264	newly 5 weeks cumul	445.	19 287.9	{812., 66 960.}	4234.62	1.16678	newly 48 weeks cumul	515.	26 578.8	{947., 97 758.}	5060.94	1.22885
18	newly 3 weeks spec	0.1	2065.11	{185., 2139.}	2.06501×10^{6}	3.77644	newly 3 weeks cumul	28.	8024.88	{1179., 9049.}	28 560.3	2.08386	newly 5 weeks spec	0.1	853.73	{40., 1950.}	853 630.	3.26684	newly 5 weeks cumul	28.	10 280.2	{1324., 11 454.}	36 615.	2.16094	newly 52 weeks cumul	28.	11 615.4	{1395., 17 769.}	41 383.6	2.20266
19	newly 3 weeks spec	126.	700.07	{0., 425.}	547.183	1.39174	newly 3 weeks cumul	683.	5164.48	{1186., 13 180.}	663.218	0.580331	newly 5 weeks spec	78.	418.11	{0., 263.}	563.679	1.61024	newly 5 weeks cumul	871.	6113.94	{1333., 18 084.}	609.92	0.528687	newly 43 weeks cumul	985.	8626.99	{1333., 27 613.}	783.577	0.537252
20	newly 3 weeks spec	0.1	2977.75	{143., 18 394.}	2.97765×10^{6}	3.75497	newly 3 weeks cumul	49.	10 746.7	{1125., 44 868.}	21 833.4	1.86561	newly 5 weeks spec	0.1	1117.9	{26., 3411.}	1.1178×10^{6}	3.15364	newly 5 weeks cumul	49.	13 884.2	{1307., 70 351.}	28 236.4	1.92766	newly 52 weeks cumul	49.	15 053.2	{1379., 75 030.}	30 622.2	1.9535
21	newly 3 weeks spec	25.	684.81	{0., 469.}	2738.84	1.7863	newly 3 weeks cumul	110.	4651.16	{1246., 4790.}	4129.65	1.29338	newly 5 weeks spec	10.	377.56	{0., 248.}	3777.6	1.6503	newly 5 weeks cumul	146.	5485.39	{1475., 9691.}	3658.6	1.2326	newly 52 weeks cumul	154.	7470.17	{1477., 12 974.}	4752.27	1.26347
22	newly 3 weeks spec	390.	695.5	{0., 884.}	216.272	1.68335	newly 3 weeks cumul	1699.	9768.25	{2333., 9650.}	484.641	0.501167	newly 5 weeks spec	240.	197.75	{0., 108.}	155.396	2.42327	newly 5 weeks cumul	2286.	10 315.8	{2357., 10 463.}	362.728	0.405245	newly 42 weeks cumul	2969.	10 596.3	{2362., 10 463.}	270.263	0.319008
23	newly 3 weeks spec	51.	3225.01	{213., 12 967.}	6229.78	1.39303	newly 3 weeks cumul	271.	17 123.7	{4710., 60 581.}	6221.17	1.48133	newly 5 weeks spec	0.1	1309.54	{14., 4455.}	1.30944×10^{6}	3.34033	newly 5 weeks cumul	288.	20 418.9	{5377., 75 331.}	6992.3	1.52465	newly 47 weeks cumul	288.	23 207.	{5692., 85 306.}	7959.57	1.56811
24	newly 3 weeks spec	1.	1491.9	{81., 1303.}	149 090.	2.71749	newly 3 weeks cumul	56.	9774.48	{3727., 7570.}	17 354.4	2.0298	newly 5 weeks spec	0.1	419.48	{2., 561.}	419 394.	2.95427	newly 5 weeks cumul	56.	10 962.4	{4047., 8926.}	19 475.6	2.07827	newly 51 weeks cumul	56.	11 571.9	{4142., 11 223.}	20 564.2	2.10101
25	newly 3 weeks spec	333.	238.16	{0., 596.}	95.8378	1.27467	newly 3 weeks cumul	1455.	6395.55	{4409., 7303.}	339.807	0.584037	newly 5 weeks spec	128.	29.28	{0., 103.}	80.3438	2.03161	newly 5 weeks cumul	1803.	6504.9	{4428., 7503.}	261.852	0.502497	newly 42 weeks cumul	2085.	6523.03	{4428., 7505.}	214.57	0.444411
26	newly 3 weeks spec	134.	1535.7	{2., 2940.}	1138.07	1.01596	newly 3 weeks cumul	702.	8955.48	{1054., 10 073.}	1183.29	0.870716	newly 5 weeks spec	48.	681.33	{0., 2300.}	1443.1	1.87691	newly 5 weeks cumul	832.	10 640.6	{1195., 14 919.}	1187.17	0.849787	newly 44 weeks cumul	1092.	12 440.7	{1380., 22 293.}	1048.76	0.792857
27	newly 3 weeks spec	208.	536.87	{2., 495.}	240.466	0.858123	newly 3 weeks cumul	1021.	8738.31	{3054., 6045.}	756.602	0.640283	newly 5 weeks spec	93.	63.45	{0., 89.}	112.355	1.50057	newly 5 weeks cumul	1280.	8975.89	{3092., 7365.}	602.555	0.556697	newly 42 weeks cumul	1520.	9024.17	{3096., 8103.}	495.432	0.487721
28	newly 3 weeks spec	419.	643.82	{0., 808.}	184.535	1.98509	newly 3 weeks cumul	1620.	8054.12	{588., 26 065.}	418.499	0.459101	newly 5 weeks spec	296.	308.91	{0., 657.}	154.963	2.10753	newly 5 weeks cumul	2248.	8837.92	{707., 26 065.}	319.944	0.408153	newly 43 weeks cumul	3114.	9511.17	{797., 26 065.}	245.063	0.398224
29	newly 3 weeks spec	193.	1904.78	{6., 6801.}	931.451	0.873649	newly 3 weeks cumul	755.	8834.77	{620., 18 967.}	1074.8	0.88425	newly 5 weeks spec	61.	1418.47	{0., 3142.}	2308.61	1.49816	newly 5 weeks cumul	915.	11 836.9	{620., 34 293.}	1201.3	0.90696	newly 45 weeks cumul	1584.	19 000.9	{620., 49 287.}	1113.38	0.837317
30	newly 3 weeks spec	34.	1844.3	{74., 5771.}	5324.41	1.27336	newly 3 weeks cumul	205.	11 638.6	{1491., 23 329.}	5577.38	1.37786	newly 5 weeks spec	6.	1179.58	{1., 2935.}	19 587.7	1.62556	newly 5 weeks cumul	220.	14 217.9	{1856., 39 014.}	6362.69	1.44009	newly 47 weeks cumul	225.	19 752.9	{2159., 85 181.}	8679.06	1.51941
31	newly 3 weeks spec	208.	2942.37	{7., 12 956.}	1372.44	0.871819	newly 3 weeks cumul	1042.	18 908.9	{1383., 57 130.}	1722.95	0.877104	newly 5 weeks spec	80.	850.49	{0., 4381.}	1069.64	1.35598	newly 5 weeks cumul	1231.	21 314.6	{1389., 75 633.}	1640.61	0.837949	newly 44 weeks cumul	1394.	22 342.5	{1389., 75 693.}	1512.69	0.799842
32	newly 3 weeks spec	553.	276.02	{0., 560.}	102.593	1.84269	newly 3 weeks cumul	1991.	9754.33	{2886., 5224.}	395.74	0.360542	newly 5 weeks spec	288.	42.9	{0., 89.}	97.2986	2.49402	newly 5 weeks cumul	2687.	9886.75	{2922., 5224.}	274.271	0.249406	newly 42 weeks cumul	3562.	9958.34	{2942., 5224.}	195.588	0.183514
33	newly 3 weeks spec	0.1	2447.69	{118., 5006.}	2.44759×10^{6}	3.67965	newly 3 weeks cumul	6.	8968.89	{689., 13 257.}	149 382.	2.68029	newly 5 weeks spec	0.1	1031.57	{13., 3234.}	1.03147×10^{6}	3.15702	newly 5 weeks cumul	6.	11 678.7	{802., 22 285.}	194 545.	2.762	newly 52 weeks cumul	6.	13 406.8	{849., 31 965.}	223 347.	2.81458
34	newly 3 weeks spec	24.	2374.78	{223., 6078.}	9794.92	1.65245	newly 3 weeks cumul	66.	8382.87	{1915., 16 557.}	12 601.3	1.89214	newly 5 weeks spec	13.	1832.93	{35., 7794.}	14 002.5	1.5883	newly 5 weeks cumul	105.	12 315.6	{2606., 29 936.}	11 629.2	1.82247	newly 48 weeks cumul	110.	21 685.3	{2674., 87 852.}	19 613.9	1.97429
35	newly 3 weeks spec	0.1	1377.13	{0., 876.}	1.3771×10^{6}	2.16868	newly 3 weeks cumul	0.1	12 120.4	{246., 37 714.}	1.21203×10^{7}	4.46016	newly 5 weeks spec	0.1	587.99	{0., 450.}	587 986.	1.61747	newly 5 weeks cumul	0.1	13 603.6	{306., 54 937.}	1.36035×10^{7}	4.50982	newly 52 weeks cumul	0.1	14 668.6	{370., 54 977.}	1.46685×10^{7}	4.54678
36	newly 3 weeks spec	107.	2474.46	{34., 4892.}	2248.47	0.711596	newly 3 weeks cumul	314.	9525.6	{263., 20 382.}	2947.1	0.851095	newly 5 weeks spec	47.	806.95	{9., 2785.}	1677.85	0.706657	newly 5 weeks cumul	444.	11 813.4	{297., 22 993.}	2580.44	0.806552	newly 52 weeks cumul	542.	12 791.5	{306., 28 752.}	2284.96	0.787815
37	newly 3 weeks spec	66.	1973.7	{180., 5365.}	2890.97	1.09843	newly 3 weeks cumul	452.	14 528.8	{7348., 18 072.}	3114.33	1.42578	newly 5 weeks spec	38.	1490.92	{2., 1726.}	3905.53	1.3543	newly 5 weeks cumul	556.	17 644.1	{9461., 38 038.}	3073.4	1.41868	newly 45 weeks cumul	581.	25 055.2	{12 069., 69 113.}	4212.43	1.50071
38	newly 3 weeks spec	0.1	913.63	{17., 1164.}	913 530.	3.2459	newly 3 weeks cumul	6.	5445.59	{108., 14 718.}	90 659.8	2.33148	newly 5 weeks spec	0.1	243.47	{4., 282.}	243 370.	2.59211	newly 5 weeks cumul	6.	6162.3	{125., 15 935.}	102 605.	2.37866	newly 52 weeks cumul	6.	6415.08	{140., 16 233.}	106 818.	2.39523
39	newly 3 weeks spec	62.	1307.74	{60., 2715.}	2014.68	1.08194	newly 3 weeks cumul	248.	9908.72	{1737., 15 726.}	3895.45	1.32293	newly 5 weeks spec	8.	446.81	{2., 750.}	5508.38	1.53159	newly 5 weeks cumul	278.	11 079.3	{1742., 18 815.}	3885.34	1.34	newly 49 weeks cumul	290.	12 027.1	{1742., 20 775.}	4047.26	1.37019
40	newly 3 weeks spec	1.	1645.79	{163., 1412.}	164 479.	2.85885	newly 3 weeks cumul	22.	9572.97	{3902., 6382.}	43 413.5	2.41246	newly 5 weeks spec	0.1	576.21	{5., 619.}	576 110.	3.19922	newly 5 weeks cumul	22.	11 106.4	{4266., 7787.}	50 383.5	2.47374	newly 51 weeks cumul	22.	12 155.9	{4370., 9103.}	55 154.1	2.50571
41	newly 3 weeks spec	9.	641.01	{0., 822.}	7054.33	1.46264	newly 3 weeks cumul	150.	11 248.	{269., 11 806.}	7398.65	1.17789	newly 5 weeks spec	0.1	155.41	{0., 254.}	155 342.	2.21168	newly 5 weeks cumul	158.	11 698.	{280., 12 807.}	7303.78	1.19372	newly 52 weeks cumul	158.	11 964.8	{292., 19 347.}	7472.65	1.21049
42	newly 3 weeks spec	40.	951.8	{18., 1796.}	2299.3	1.07551	newly 3 weeks cumul	178.	5901.32	{566., 6987.}	3215.35	1.23843	newly 5 weeks spec	2.	405.9	{0., 1006.}	20 225.	1.81882	newly 5 weeks cumul	187.	6911.7	{567., 9297.}	3596.1	1.27746	newly 49 weeks cumul	187.	7857.05	{567., 11 846.}	4101.63	1.31792
43	newly 3 weeks spec	34.	2462.72	{164., 9067.}	7143.94	1.19093	newly 3 weeks cumul	177.	11 338.3	{1199., 16 574.}	6308.03	1.2971	newly 5 weeks spec	7.	826.49	{30., 3007.}	11 707.	1.2747	newly 5 weeks cumul	204.	13 625.8	{1323., 37 549.}	6580.4	1.29078	newly 52 weeks cumul	209.	14 676.3	{1360., 43 233.}	6922.38	1.3026
44	newly 3 weeks spec	5.	1109.99	{0., 416.}	22 125.8	1.65942	newly 3 weeks cumul	94.	3592.99	{273., 2309.}	3722.33	1.13184	newly 5 weeks spec	0.1	932.19	{0., 188.}	932 118.	2.49743	newly 5 weeks cumul	94.	5665.99	{339., 2707.}	5927.65	1.21077	newly 51 weeks cumul	94.	7155.03	{385., 2890.}	7511.73	1.2658
45	newly 3 weeks spec	93.	932.74	{25., 1276.}	937.14	0.547632	newly 3 weeks cumul	558.	3232.05	{390., 3625.}	495.281	0.474104	newly 5 weeks spec	65.	801.31	{0., 1402.}	1216.23	0.936211	newly 5 weeks cumul	698.	4926.76	{498., 6271.}	620.461	0.464683	newly 47 weeks cumul	726.	8003.74	{604., 17 835.}	1014.38	0.531553
46	newly 3 weeks spec	37.	3014.95	{71., 9904.}	8050.03	1.40365	newly 3 weeks cumul	384.	8903.77	{356., 34 000.}	2227.24	1.0208	newly 5 weeks spec	4.	2269.57	{28., 12 888.}	56 639.3	2.10466	newly 5 weeks cumul	404.	13 898.7	{442., 55 479.}	3346.92	1.12647	newly 48 weeks cumul	409.	20 973.1	{489., 90 211.}	5033.43	1.25649
47	newly 3 weeks spec	33.	2614.51	{0., 5376.}	7880.76	2.03665	newly 3 weeks cumul	146.	14 990.4	{3062., 23 828.}	10 169.	1.74454	newly 5 weeks spec	17.	2121.5	{0., 3047.}	12 437.4	2.07853	newly 5 weeks cumul	191.	19 518.8	{4043., 52 260.}	10 121.	1.76419	newly 48 weeks cumul	201.	27 274.4	{6271., 91 492.}	13 471.1	1.90466
48	newly 3 weeks spec	101.	679.69	{0., 891.}	660.485	1.59065	newly 3 weeks cumul	455.	4258.89	{1733., 4249.}	836.02	0.846369	newly 5 weeks spec	16.	562.44	{0., 742.}	3517.63	1.55171	newly 5 weeks cumul	527.	5431.5	{1758., 5508.}	930.645	0.831167	newly 43 weeks cumul	536.	8805.83	{1773., 17 614.}	1542.88	0.881469
49	newly 3 weeks spec	21.	1932.46	{164., 2584.}	9102.19	1.5867	newly 3 weeks cumul	128.	8126.21	{667., 11 202.}	6248.6	1.41888	newly 5 weeks spec	0.1	946.14	{90., 1496.}	946 040.	3.63943	newly 5 weeks cumul	131.	10 425.8	{880., 13 962.}	7858.59	1.53442	newly 49 weeks cumul	131.	13 823.3	{1238., 33 442.}	10 452.1	1.68925
50	newly 3 weeks spec	8.	1392.08	{4., 3726.}	17 319.5	1.72067	newly 3 weeks cumul	96.	10 330.	{2149., 44 077.}	10 662.	1.66716	newly 5 weeks spec	0.1	482.82	{0., 860.}	482 776.	2.55626	newly 5 weeks cumul	102.	11 489.6	{2712., 48 484.}	11 165.8	1.7326	newly 50 weeks cumul	102.	15 779.7	{3146., 67 985.}	15 371.7	1.8686
